# Machine learning and deep learning frameworks for the automated analysis of pain and opioid withdrawal behaviors

**DOI:** 10.3389/fnins.2022.953182

**Published:** 2022-09-26

**Authors:** Jacob R. Bumgarner, Darius D. Becker-Krail, Rhett C. White, Randy J. Nelson

**Affiliations:** Department of Neuroscience, Rockefeller Neuroscience Institute, West Virginia University, Morgantown, WV, United States

**Keywords:** pain, opioid withdrawal, opioid use disorder (OUD), deep learning, machine learning, markerless tracking, automated behavioral analysis, pose estimation

## Abstract

The automation of behavioral tracking and analysis in preclinical research can serve to advance the rate of research outcomes, increase experimental scalability, and challenge the scientific reproducibility crisis. Recent advances in the efficiency, accuracy, and accessibility of deep learning (DL) and machine learning (ML) frameworks are enabling this automation. As the ongoing opioid epidemic continues to worsen alongside increasing rates of chronic pain, there are ever-growing needs to understand opioid use disorders (OUDs) and identify non-opioid therapeutic options for pain. In this review, we examine how these related needs can be advanced by the development and validation of DL and ML resources for automated pain and withdrawal behavioral tracking. We aim to emphasize the utility of these tools for automated behavioral analysis, and we argue that currently developed models should be deployed to address novel questions in the fields of pain and OUD research.

## Introduction

The ongoing opioid epidemic is currently the worst it has been in the history of the United States. Exacerbated by the global coronavirus disease 2019 (COVID-19) pandemic, in 2021 the U.S. saw the highest annual drug overdose death toll ever recorded, with an estimated 107,622 overdose deaths (Ahmad et al., 2021). Of these deaths, 75% involved opioid use (Ahmad et al., 2021). Among other factors, one major contribution to the opioid epidemic is the prevalence of chronic pain (Volkow and McLellan, 2016). Because of the lack of widespread non-opioid analgesics and alternative therapeutic options, opioids are continually prescribed for chronic pain management, despite the number of associated risk factors (Nadeau et al., 2021). As a result of this factor and increasing accessibility to potent synthetic opioids, including fentanyl, there is an ever-growing need to identify treatment strategies that can mitigate this ongoing crisis (Skelly et al., 2020).

Translational models of pain and opioid withdrawal behaviors are crucial for the development of safer non-opioid analgesics and other treatment options for opioid use disorders (OUDs). A natural aspect of these preclinical experiments is the characterization of behavioral responses in translational models, particularly rodent models, which to date has primarily relied on human visual perception. As such, current behavioral paradigms in pain or opioid withdrawal research in rodents still rely on manual scoring (Deuis et al., 2017; Bravo et al., 2020, 2021). This process is often time-consuming, labor-intensive, and error-prone, even when conducted by trained researchers. The current approach of manual scoring also has limited scalability and introduces potential contributions to the reproducibility crisis.

Recent advances in the efficiency and accessibility of deep learning (DL) and machine learning (ML) frameworks have provided a clear route to challenge the issues associated with manual scoring of pain behavior (Mathis and Mathis, 2020). Widely accessible open-source tools being developed and used in preclinical research are now capable of automatically labeling and quantifying complex behaviors with accuracy that can match human performance (Table 1; Sturman et al., 2020b). By addressing the challenges associated with manual scoring, accurate automated behavioral analysis tools that leverage the utility of ML and DL frameworks can improve the rigor and throughput of preclinical and ecological research (Anderson and Perona, 2014). Moreover, these tools can provide improved insight into naturalistic behaviors that may otherwise go unnoticed by human observers (Datta et al., 2019). In this perspective, we outline the current ML and DL frameworks being used to label behavior in translational models and look ahead at how these tools could be applied to accelerate the preclinical study of pain and OUD.

**TABLE 1:** Summary of automated pain and opioid use disorder (OUD) behavioral analysis articles.

Citation	Relevant field	Behaviors tracked	Frameworks and software used	Repository and trained model links
Abdus-Saboor et al., 2019	Pain	Paw and face labeling	PainAssaySVM	https://github.com/longdecision/PainAssaySVM
Jones et al., 2020	Pain	Peak paw withdrawl height and guarding duration	SLEAP, PAWS, and ProAnalyst	https://github.com/crtwomey/paws
Bohic et al., 2021	Pain	Allodynia and others	DeepLabCut, B-SOiD, MoSeq, PAWS, pybasicbayes, scikit-learn, and Gensim	NA
Kopaczka et al., 2018	Pain	Mouse grimace scale	PyTorch	NA
Andresen et al., 2020	Pain	Postoperative pain	TensorFlow, OpenCV	NA
Tuttle et al., 2018	Pain	Pain or no pain	TensorFlow	NA
Kobayashi et al., 2021	Pain and OUD	Scratching	Keras, OpenCV	NA
Wotton et al., 2020	Pain and OUD	Licking/non-licking events such as paw flick	DeepLabCut, MatLab for GentleBoost model, and k-nearest neighbor classifier.	https://www.kumarlab.org/2020/10/05/machine-learning-based-automated-phenotyping-of-inflammatory-nocifensive-behavior-in-mice/
Kopaczka et al., 2019	Pain and OUD	General movement, grooming, and resting	NA	NA
Dao et al., 2021	Pain and OUD	Rat ultrasonic vocalizations	DeepSqueak	NA
Murphy et al., 2021	OUD	Jumping, rearing, grooming, tremors, etc.	DeepLabCut, SimBA,	NA

## Artificial intelligence as a tool for behavioral analysis

**FIGURE 1:**
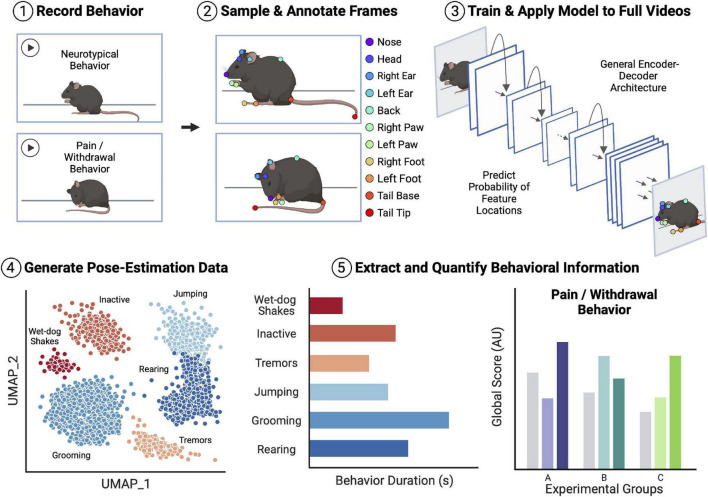
Overview of an example workflow for deep learning (DL)-assisted automated pain/withdrawal behavioral analysis. Animal behavior is first recorded on video, including both neurotypical behavior and potential pain/withdrawal behavior. Distinct frames are then sampled from the video pool, and the points of interest in the frames are manually annotated. The labeled frames are then used to train an encoder-decoder convolutional neural network (CNN) with tools such as DeepLabCut or SLEAP. Once the model is trained and achieves a desired level of accuracy, the full videos are fed into the model to generate pose-estimation data for all mice. Finally, behavioral information is extracted from the estimated pose data and quantified ahead of statistical comparisons. This extracted behavioral information can then be fed into field-standard global scoring algorithms and models, thus allowing for comparison of pain/withdrawal behavior between control and experimental groups of mice. Importantly, there are many easily accessible and open-source tools for each step of the example analysis pipeline, many of which have been tested and validated in the context of translational pain and opioid abuse research. Figure created with BioRender.com.

Automated behavior tracking in most experimental protocols generally follows a two-stage pipeline (Figure 1). The first stage of most of these pipelines involves video recording and subsequent point labeling/tracking of specific body parts of the studied animals. Numerous highly effective tools exist for point-tracking without added markers on the animals (i.e., markerless tracking). Examples of these tools include DeepLabCut (Mathis et al., 2018; Lauer et al., 2022), SLEAP (Pereira et al., 2022), DANNCE (Dunn et al., 2021), and Anipose (which relies on DeepLabCut) (Karashchuk et al., 2021). Broadly, these markerless point estimation tools use convolutional neural network (CNN) models with encoder-decoder architectures to create probability density plots for each trained feature. Each pixel value in the output plots represents the probability of the presence of the feature of interest. The probability densities are then used to localize points within the images. Following the identification of the desired points, whole-body or whole-limb point skeletons can be constructed for subsequent pose estimation and behavioral classifications. Moreover, several point estimation models can produce point labels and point skeletons for multiple animals within the same video recording (Lauer et al., 2022; Pereira et al., 2022).

The second stage of automated behavioral analysis involves the process of extracting meaningful information from the labeled point data (Sturman et al., 2020a). The approaches in this stage are much more varied and specific to the desired type of behavioral analysis being conducted. Many of the tools used in this stage of analysis rely on traditional tracking algorithms as well as supervised and unsupervised ML and DL. Basic behaviors, such as total movement, zone entries, and even paw flicks, can be tracked and quantified with traditional algorithms. In contrast, the identification of more abstract or nuanced behaviors, such as grooming, shaking, licking, and biting, require the use of more complicated algorithms [e.g., UMAP (McInnes et al., 2018)], ML algorithms (e.g., Random forest classifiers), and DL models (e.g., convolutional recurrent neural networks). Tools used in this domain to extract and quantify pose and behavioral information include SiMBA (Nilsson et al., 2020), B-SOiD (Hsu and Yttri, 2021), MoSeq (Wiltschko et al., 2015, 2020), and uBAM (Brattoli et al., 2021), among others (Sturman et al., 2020a; Hu et al., 2022).

Importantly, numerous tools and pipelines that can be used for both stages of automated behavioral analysis have been developed and validated for automated scoring of pain and opioid withdrawal behaviors. Moreover, several of these tools are open-source and easily accessible. In the next two sections, we will discuss the development, validity, and verification of several automated behavioral analysis tools in these two fields.

## Automated analysis of pain behavior

Pain is a highly subjective experience that involves both affective and sensory-discriminative neurocircuitry engagement (Baliki and Apkarian, 2015). Preclinical research relies on the visual and sometimes acoustic observation of behavioral alterations in non-human animals to determine the presence of pain. Without using automated analysis techniques, pain scoring typically involves real-time visual inspections and timings of discrete behavioral events, such as paw flicking, extended paw withdrawals, jumping, grooming, licking/biting, and even squeaking (Deuis et al., 2017). Behavioral testing sessions are also frequently recorded for subsequent timing and count verifications or for delayed behavioral scoring.

Both real-time and recording-based behavioral scoring are highly subjective skills that require expert training and careful, rigorous attention. Real-time behavioral observations can suffer from experimenter fatigue, impaired viewing angles, and differing opinions over discrete events. Recording-based observations offer improvements over real-time scoring, as behavioral observations can be more carefully confirmed with repeated inspections, provided that the multiple viewing angles and high frame rates are used to prevent missed or obscured behavior by animal movement or crouching. However, this latter approach can involve long hours of staring at screens, which can lead to experimenter fatigue and potential inaccuracies. With careful recording setups and pipeline validation, automated analysis techniques offer the ability to overcome several of these issues (Fried et al., 2020). Indeed, numerous models have been developed to automate the analysis of scratching, paw withdrawal, general pain states, and mouse-grimace scale scoring.

Several automated models have been independently developed to identify scratching and hind paw flicking/withdrawal behaviors. One model used a convolutional recurrent neural network that relies on an extended temporal window of behavioral information to determine whether scratching was occurring in any given frame (Kobayashi et al., 2021). The authors reported an overall 94.8% accuracy of scratching detection and validated the automated detection in a dinitrofluorobenzene-induced dermatitis experiment (Kobayashi et al., 2021). Another group used DeepLabCut to track paw movement from a bottom-up view and the GentleBoost classifier to label licking/non-licking behavioral events (Wotton et al., 2020). This model had a 98% accuracy in second-by-second behavioral classification, and the practical performance of this model was validated with analgesic and formalin injections (Wotton et al., 2020). Lastly, a third group developed an ML-based analysis pipeline that uses manual paw and face labeling to generate a rigorous pain score (Abdus-Saboor et al., 2019). The pipeline reduces paw and facial movement feature dimensionality with principal component analyses into principal component scores, which are fed into a trained support vector machine (SVM) that provides a single pain metric. Importantly, this approach could be further automated in the future by using point-labeling models to automatically track paw and facial movement (Abdus-Saboor et al., 2019).

Indeed, the same group from the previous study later introduced two updated pain analysis pipelines that automatically track paw and body movement and then extract and analyze the features of the movement. In the first updated pipeline, SLEAP was used to track paw movement and then various algorithms were used to extract information from the paw movement, including peak paw withdrawal height, and guarding duration (Jones et al., 2020). Then, a univariate pain score is generated using ordinal logistic regression. This first pipeline was validated with the presentation of different innocuous and noxious stimuli and the chemogenic activation of the pain assembly in the basolateral amygdala (Jones et al., 2020). In the second updated pipeline, the same group uses a wide group of tools and algorithms, including high speed behavioral tracking, time-of-flight infrared tracking, DeepLabCut, principal component analysis, B-SOiD, MoSeq, and learned embedding with doc2vec, to automatically extract and quantify allodynia and other pain behaviors in mice (Bohic et al., 2021).

Another line of work has sought to automate the application of the mouse grimace scale (MGS), which uses a collection of five facial features to characterize the presence of pain (Langford et al., 2010). Because MGS scoring requires trained individuals to provide the analyses, automation of this scoring was well-needed (Kopaczka et al., 2018). In the first example of automated MGS analysis, one group used a U-Net architecture to segment the regions of interest, including the ears, eyes, and body. The regions of interest are then fed into a CNN that outputs to a single unit with a linear regression classifier to label input images with 0–9 grimace scores (based on the 0–1 regression output) (Kopaczka et al., 2018). Moreover, the authors provide a GUI and server-client architecture to access automated and real-time tracking of behavioral experiments. The model that they developed had a 0.871 mean absolute error on their 0–9 scale (Kopaczka et al., 2018). A second group trained a CNN with MGS labels to identify the presence of pain (Andresen et al., 2020). With accuracy levels approaching >90%, the group was able to accurately identify the presence of postoperative pain in mice (Andresen et al., 2020). Lastly, another group used transfer learning from an InceptionV3 architecture that was pretrained on ImageNet (Tuttle et al., 2018). The final FC and two Softmax layers were retrained to provide “pain”/“no pain” confidence scores. When analyzing their entire test image set, the model had a reported accuracy of 84%.

## Automated analysis of opioid withdrawal behavior

Opioid use disorder is a chronic debilitating condition driven by a cycle of compulsive opioid use, withdrawal, craving, and relapse (Strang et al., 2020). Importantly, opioid dependence and opioid withdrawal syndrome are fundamental features of OUD. These features are thought to induce long-term behavioral and physiological changes as well as increase motivation and propensity for future opioid abuse (Strang et al., 2020). In preclinical research, opioid dependence, and withdrawal are key aspects of modeling OUD. In rodents, opioid dependence is generally achieved through either continual access (e.g., volitional self-administration) or through a paradigm of escalating doses of injections (i.e., non-volitional exposure) (Spanagel, 2022). Opioid dependence in rodents has been shown to produce a range of withdrawal symptoms, including increased grooming, wet-dog shakes, tremors, jumping, rearing, wall climbing, diarrhea, and anxiety-like behavior, among others (Bravo et al., 2020, 2021; Gipson et al., 2020). Along with a withdrawal-like syndrome, opioid dependence in rodents also induces opioid-induced hyperalgesia (Liang et al., 2006; Roeckel et al., 2016).

As with pain behavior scoring, manual scoring of rodent withdrawal behavior involves visual inspection and timing of discrete behavioral events (e.g., grooming, wet-dog shakes, tremors, etc.). Given the drawbacks of manual scoring, automated analysis techniques offer the ability to increase efficiency and decrease error in the assessment of opioid withdrawal in preclinical models of OUD (Fried et al., 2020). However, there has been a remarkable absence of automated analysis in this context. Notably, only one identified study focused on the automated analysis of opioid withdrawal behavior. In this preliminary study, Murphy and colleagues set out to establish a translational model of oral oxycodone self-administration using automated and open-source tools (Murphy et al., 2021). Specifically, markerless pose estimation and subsequent supervised ML predictive classifiers for opioid withdrawal-related behavior were used in C57BL/6J mice that were trained to self-administer oxycodone orally for 12 days with *ad libitum* access. DeepLabCut was used for markerless pose estimation of head, paw, torso, and tail movements. Quantification and extraction of behavioral information were subsequently performed using SimBA, thus allowing Murphy and colleagues to assess jumping, climbing, rearing, grooming, tremors, and Euclidean distance of displacement of all body parts. Ultimately, this automated withdrawal behavioral analysis pipeline was able to detect and quantify physical signs of dependence that are consistent with opioid withdrawal syndrome in mice (Murphy et al., 2021).

To our knowledge only a single paper has been published that explicitly focused on the automated analysis of opioid withdrawal behaviors; however, other pipelines have been built to track behaviors that are related to opioid withdrawal (Kopaczka et al., 2019). For example, as described, one group built a two-stream model with the ResNet101 architecture to classify general activity patterns in mice, including general movement, grooming, resting, turning, and facing away from the camera (Kopaczka et al., 2019). Importantly, the grooming and movement classifications from this model could be generalized and applied to opioid abuse research. Interestingly, another study automated the analysis of rat ultrasonic vocalizations during a chronic fentanyl self-administration paradigm (Dao et al., 2021). This was achieved using DeepSqueak, a DL-based system for analyzing ultrasonic vocalizations (Coffey et al., 2019). Given that ultrasonic vocalizations are thought to correlate with rodent affect, this tool could be valuable in analyzing another translational aspect of withdrawal syndrome. Finally, other models described in the previous sections, such as the paw flick (Wotton et al., 2020) or scratching (Kobayashi et al., 2021) classification models could also be generalized and used in the context of opioid dependence and withdrawal.

## Limitations

As no tool is perfect, it is important to note that these automated pipelines have their own sets of pitfalls, in common with manual scoring. Automated models still require manual labeling of training data (although frankly minimal, e.g., 100–150 frames for accurate DeepLabCut models). The frames that are automatically labeled and automated analysis frameworks must also be manually validated, which means there is still a need for local expertise over the behaviors that are being automatically scored. Moreover, many of these pipelines require a certain degree of technical and computational proficiency and technological specifications. In terms of confirming model tracking and analysis accuracy, an additional limitation is that there is currently no unified database of labeled pain or OUD behavioral recordings that can be used for model validations. Nonetheless, automated behavioral tracking and analysis pipelines are being actively improved, and their associated pitfalls are being minimized. In that regard, we argue that it is important for the developers of automated behavioral analysis tools to consider the end-user in mind when creating and deploying automated analysis packages. Future tools should be open-source and easily accessible in public codebase repositories, and tools should ideally have high-level APIs or even GUIs for accessible deployment.

## Future directions

Automated behavioral tracking and analysis tools are well-positioned to blossom in the fields of pain and opioid abuse research. We argue that the accuracy and validation testing of these tools have demonstrated their feasibility in the contexts of pain and OUD research. Particularly in the context of pain research, the articles described in this review have demonstrated the potential to classify and quantify several nuanced behaviors with high levels of accuracy. Preliminary work in the context of OUD has demonstrated similar effectiveness of DL and ML tools for automated scoring of withdrawal-related behaviors in mice. These already highly accurate and primarily open-source automated pipelines discussed here will act as the foundation for a future of automated pain and OUD behavioral testing (Supplementary Table 1).

Taken together, ML and DL automated behavioral analysis pipelines have proven to be highly powerful across many fields. With their increased implementation, automated ML and DL-based automated behavioral analysis have the potential to increase the efficiency of pre-clinical investigation of pain and opioid abuse, as well as increase its rigor and reproducibility. Now is the time for scientists working in the fields of pain and OUD research to move beyond the validation stages and begin implementing automated behavioral analysis tools to test novel research questions. In doing so, researchers may be afforded an opportunity to ask more complex and comprehensive questions in their pursuit of novel pain therapeutics with less abuse potential.

## Author contributions

JB, DB-K, and RN designed, wrote, and edited the manuscript. RW helped with research and writing of the manuscript. DB-K and JB designed the figure. All authors contributed to the article and approved the submitted version.
