# Supplementary material for: Machine learning and deep learning frameworks for the automated analysis of pain and opioid withdrawal behaviors
Source: Front Neurosci. 2022 Sep 26;16:953182. doi: 10.3389/fnins.2022.953182 (PMC9549170; doi:10.3389/fnins.2022.953182)
Supplement: Supplementary file 1 [file Table_1.DOCX]

| Supplemental Table 1 – Open-Source Resources for Automated Behavioral Analysis | | | |
| --- | --- | --- | --- |
| **Framework Name** | **Functions** | **Citation** | **Repository** |
| DeepLabCut | Multi-animal point tracking & pose estimation | [Mathis et al. (2018)](https://www.nature.com/articles/s41593-018-0209-y)  [Lauer et al. (2022)](https://www.nature.com/articles/s41592-022-01443-0) | <https://github.com/DeepLabCut/DeepLabCut> |
| SLEAP | Multi-animal point tracking & pose estimation | [Pereira et al. (2018)](https://www.nature.com/articles/s41592-018-0234-5)  [Pereira et al. (2022)](https://www.nature.com/articles/s41592-022-01426-1) | <https://github.com/talmolab/sleap> |
| Anipose | Point tracking & pose estimation | [Karashchuk et al. (2021)](https://www.sciencedirect.com/science/article/pii/S2211124721011797) | <https://github.com/lambdaloop/anipose> |
| DeepPoseKit | Point tracking & pose estimation | [Graving et al. (2019)](https://elifesciences.org/articles/47994) | <https://github.com/jgraving/DeepPoseKit> |
| MoSeq | 3D point tracking & predictive behavioral classification | [Wiltschko et al. (2020)](https://www.nature.com/articles/s41593-020-00706-3) | <https://github.com/dattalab/moseq-drugs> |
| DeepSqueak | Ultrasonic vocalization detection & classification | [Coffey et al. (2019)](https://www.nature.com/articles/s41386-018-0303-6) | <https://github.com/DrCoffey/DeepSqueak> |
| SimBA | Point inference & predictive behavioral classification | [Nilsson et al. (2020)](https://www.biorxiv.org/content/10.1101/2020.04.19.049452v2) | <https://github.com/sgoldenlab/simba> |
| B-SOID | Point inference & predictive behavioral classification | [Hsu & Yttri (2021)](https://www.nature.com/articles/s41467-021-25420-x) | <https://github.com/YttriLab/B-SOID> |
| uBAM | Point inference, predictive behavioral classification, & deviation magnification | [Brattoli et al. (2021)](https://www.nature.com/articles/s42256-021-00326-x) | <https://github.com/utabuechler/uBAM> |
| LabGym | Multi-Animal-Tracking and Behavioral Classification and Quantification | [Hu et al. (2022)](https://www.biorxiv.org/content/10.1101/2022.02.17.480911v3) | <https://github.com/umyelab/LabGym> |
| MotionMapper | Point inference & predictive behavioral classification for *Drosophila melanogaster* | [Berman et al. (2014)](https://royalsocietypublishing.org/doi/10.1098/rsif.2014.0672) | <https://github.com/gordonberman/MotionMapper> |
| DANNCE | Whole-body 3D point tracking from multiangle cameras | [Dunn & Marshall et al. (2021)](https://www.nature.com/articles/s41592-021-01106-6#Sec12) | <https://github.com/spoonsso/dannce> |
